# Aseptic midterm survival rates between different cemented tibial stem designs in hinged total knee arthroplasty: a 6-year evaluation from the German Arthroplasty Registry

**DOI:** 10.1007/s00402-024-05273-x

**Published:** 2024-04-16

**Authors:** Alexander Maslaris, Alexander Grimberg, Oliver Melsheimer, Elefterios Tsiridis, Georg Matziolis

**Affiliations:** 1 Orthopaedic Department at Campus Eisenberg, University Hospital Jena, Klosterlausnitzer Str. 81, 07607 Eisenberg, Germany; 2 German Arthroplasty Registry gGmbH (EPRD), EPRD Deutsche Endoprothesenregister gGmbH, Straße des 17. Juni 106-108 (Eingang Bachstraße), 10623 Berlin, Germany; 3 Academic Orthopaedic Department, Papageorgiou General Hospital, Aristotle University Medical School, Thessaloníki, Greece

**Keywords:** Aseptic survival rate, Cumulative aseptic revision rate, Cemented stems, Tibial stems, Stem conicity, Conical stems, Cemented revision total knee arthroplasty, Stem length, Stem diameter, Stem offset, Stem design

## Abstract

**Introduction:**

The rate of revision TKA and thus the use of hinged implants (HI) steadily rises. Aseptic loosening lies on the top of the failure patterns. However, no evidence exists until now based on national scale high-caseloads that analyzes the impact of cemented HI stem-design on aseptic survival rates.

**Methods:**

Data on aseptic HI-revisions with full-cemented tibia-stems were conducted from the German Arthroplasty Registry. Cases were divided in primary HI (PHI) and HI used in revision operations (RHI). Endpoint was a new revision following either a PHI or an RHI. The impact of stem conicity (conical vs. cylindrical), diameter (≤ 13 mm vs. > 13 mm), length (≤ 90 mm vs. > 90 mm) and offset on the 6-Year-Cumulative-Aseptic-Revision-Rate (6Y-CARR) was estimated via Kaplan–Meier curve and compared between groups via Log-Rank-Tests.

**Results:**

3953 PHI and 2032 RHI fulfilled inclusion-criteria. Stem conicity had no impact on 6Y-CARR (p = 0.08 and p = 0.8). Diameter > 13 mm hat an impact on PHI (p = 0.05) with lower 6Y-CARR but not on RHI (p = 0.2). Length > 90 mm showed significantly worst 6Y-CARR in PHI (p = 0.0001) but not in RHI (p = 0.3). Offset-stems showed significantly better 6Y-CARR in PHI (p = 0.04), but not in RHI (p = 0.7).

**Conclusion:**

There was no significant impact of the cemented tibia-stem conicity on 6Y-CARR, neither in PHI nor in RHI. The effect of length, diameter and offset on the 6Y-CARR observed in the PHI, was not detectable in the more complex RHI-cases reflecting its limited clinical relevance by itself in more multifactorial backgrounds. Therefore, results must be interpreted with caution due to considerable system-effects and different utilization-scenarios.

## Introduction

Due to a steadily growing volume of revision total knee arthroplasty (RTKA), the number of re-revisions is consequently also rising [1–9]. Aseptic loosening of the tibia is a common failure pattern in RTKA [10–20].

Implants with comparable durability and easier reversibility are preferable, which is why cemented RTKA stems are viewed critically by some authors as removal of well-fixed stemmed implants and the surrounding cement mantel in septic cases can be challenging [16, 21–26]. Resent observations on the revisability of well-fixed cemented RTKA implant have shown that conical stems provide lower resistance to extraction than cylindrical stems and therefore are potentially associated with a more friendly revisability [27–29]. When comparing long-term outcomes between cemented and cementless RTKAs the literature provides inconclusive evidence with controversial recommendations [16, 20, 25, 26, 30–39]. However, a tendency towards slightly superior outcomes in terms of aseptic survival and loosening rates is noticeable in cemented RTKA stems [34–36, 40].

In a pilot study on modes of RTKA-failure currently published, the preliminary results revealed a higher incidence of aseptic loosening in the cemented cylindrical RTKA stems than in the cemented conical stems [18]. However, the small sample sizes and the retrospective single-center study design limit any conclusions. Thus, a higher scale study analyzing the impact of stem design also on the survival rates of RTKA implants, is necessary to highlight this topic and provide further clarification.

Furthermore, based on implant-specific survivals of established hinged RTKA systems with either conical or cylindrical stems published on national registers, there are only marginal non-specific discrepancies, making it difficult to differentiate the effect of stem conicity from other implant design properties.

Aim of the present study is therefore to initiate a large-scale analysis and at the same time to assure controlled and homogeneous sample grouping. The German Arthroplasty Registry captures almost all RTKA cases regardless of the type of hospital they were performed in [41, 42] and is able to undertake via a sophisticated algorithm several differentiations between implant design characteristics as required. Therefore, it provides a reliable source to detect possible implant-associated RTKA outcome tendencies. Thus, the EPRD database was used to examine the effect of four stem design properties: (1) conicity, (2) length, (3) diameter und (4) offset, on the risk of aseptic failure in cemented RTKA separating primary use from revision cases. Focus was given solely on tibial stems. We hypothesized that (a) RTKA with cemented conical stem extensions will have superior survival rates than with cylindrical stems and (b) the other stem design properties will have only minor influence.

## Methods

### Data collection

The German Arthroplasty Registry (Endoprothesenregister Deutschland, EPRD) collects data since 2012 as a non-profit organization founded by surgeons and the German Society of Orthopedics and Orthopedic Surgery (DGOOC) based on public health insurances, the German Medical Technology Association and hospitals that perform arthroplasties including this way approximately 70% of primary and revision TKA cases [43]. To classify and identify diagnosis and procedures accurately EPRD uses the German versions of the International Classification of Procedures in Medicine (ICPM) and the 10th International Classification of Diseases (ICD-10).

### Study Subjects

A prospective registry data analysis from the German Arthroplasty Registry EPRD was conducted. All aseptic RTKA implantations that possessed a full-cemented tibial stem extension and were performed from the 22nd of December 2010 until the 31st of September 2021 were initially detected. The starting points were divided in (1) primary arthroplasties with hinged implants (*PHI*) and (2) revision cases with hinged implants (RHI). As endpoints were considered either a 1st aseptic revision of the PHI or an aseptic re-revision of the RHI. All causes of aseptic failure that led to re-operation were identified and the 6-year commutative aseptic revision (or re-revision) rate (6Y-CARR) was determined.

The following inclusion criteria were applied:

- Solely hinged RTKA models.
- Tibial tray as monoblocs, modular, preassembled or prefixed.
- Tibial stems as monobloc or modular.
- Full-cemented tibial stem.
- Tibial stem form: straight, fluted, with or without offset, all lengths and diameters.

In order to focus mainly on failure mechanisms related to cemented stems of HI, following exclusion criteria were considered:

- Septic surgery.
- Tumor surgery.
- Cementless stem fixation.
- Bowed stems.
- Solely patella revision or patella resurfacing.

### Comparative group design

Based on the stem design properties below, following subgroups were subsumed:

1. Stem conicity: (1) conical stem designs (*Co*) vs. (2) cylindrical stem designs (*Cy*).
2. Stem diameter (applies only for *Cy* stems): (1) ø ≤ 13 mm vs. (2) ø > 13 mm.
3. Stem length: (1) ≤ 90 mm vs. (2) > 90 mm.
4. Stem Offset (applies only for *Cy* stems): (1) with offset vs. (2) without offset.

The separation of stem profile in Co and Cy stem design was based on manufacturer specifications and measurements done using the planning software MediCAD^®^. Combined stem designs with conical stem proportions ≤ 30% were included in the Cy group.

### Statistical analysis

For the statistical analysis the Software R (R Foundation for Statistical Computing, Vienna, Austria; https://www.R-project.org/) was used. To analyze the survival function on the extracted EPRD implant data the 6Y-CARR inclusive 95% confidence interval was derived from the Kaplan–Meier-estimator. The Log-rank test was used to perform comprehensive comparisons between two groups. Differences in aseptic revision rates were statistically significant if p ≤ 0.05.

## Results

An overall of 5.985 aseptic HI with cemented tibial stems met inclusion criteria. 3953 were PHI cases and 2032 were used in RHI situations. The data distribution between these two main groups, stratified additionally by its stem conicity are summarized in Table 1a–b.

**Table 1:** a–b Descriptive data set between the two main groups: (a) primary cases with hinged implants (PHI) and (b) revision cases with hinged implants (RHI)

	Stratified by stem conicity		
	Co	Cy	Sign. (p value)
(a) Primary cases (PHI)			
Cases (n)	2114	1839	
Stem profile: Cy (%)	0 (0.0)	1839 (100.0)	< 0.001
Age: mean (SD)	75.07 (8.76)	73.71 (9.66)	< 0.001
Gender: females (%)	1697 (80.3)	1516 (82.4)	0.090
With cones or sleeves: true (%)	0 (0.0)	8 (0.4)	0.007
Stem offset: without offset (%)	2114 (100.0)	478 (26.0)	< 0.001
Stem diameter: > 13 mm (%)	N/A	1028 (73.3)	N/A
Stem length: > 90 mm (%)	2025 (95.8)	338 (18.4)	< 0.001
(b) Revision cases (RHI)			
Cases (n)	647	1385	
Stem profile: Cy (%)	0 (0.0)	1385 (100.0)	< 0.001
Age: mean (SD)	73.23 (8.76)	71.55 (9.86)	< 0.001
Gender: females (%)	452 (69.9)	1070 (77.3)	< 0.001
With cones or sleeves: n (%)	35 (5.4)	44 (3.2)	0.021
Stem offset: without offset (%)	647 (100.0)	397 (28.7)	< 0.001
Stem diameter: > 13 mm (%)	N/A	691 (63.3)	N/A
Stem length: > 90 mm (%)	609 (94.1)	453 (32.7)	< 0.001

*N/A* not applicable, *Co* conical stems, *Cy* cylindrical stems

The estimated 6Y-CARR differences for all group comparisons are summarized in Table 2a–b.

**Table 2:** a–b Summery of all 6Y-CARR mean comparisons between groups

Comparisons	6Y-CARR (%)	Sign p value
(a) Primary cases (PHI)		
Overall	2.5%	
Co vs. Cy	2.7 vs. 2.3	0.08
Diameter: ≤ 13 vs. > 13 (Cy)	2.6 vs. 1	0.05
Length: ≤ 90 mm vs. > 90 mm (Cy)	1.4 vs. 6.8	< 0.001
Offset: with vs. without (Cy)	1.3 vs. 5.1	0.04
(b) Revision cases (RHI)		
Overall	14.7%	
Co vs. Cy	13.5 vs. 15.8	0.8
Diameter: ≤ 13 vs. > 13 (Cy)	13 vs. 15.1	0.2
Length: ≤ 90 mm vs. > 90 mm (Cy)	13 vs. 14.8	0.3
Offset: with vs. without (Cy)	14 vs. 12.2	0.7

### Stem conicity

**Fig. 1:**
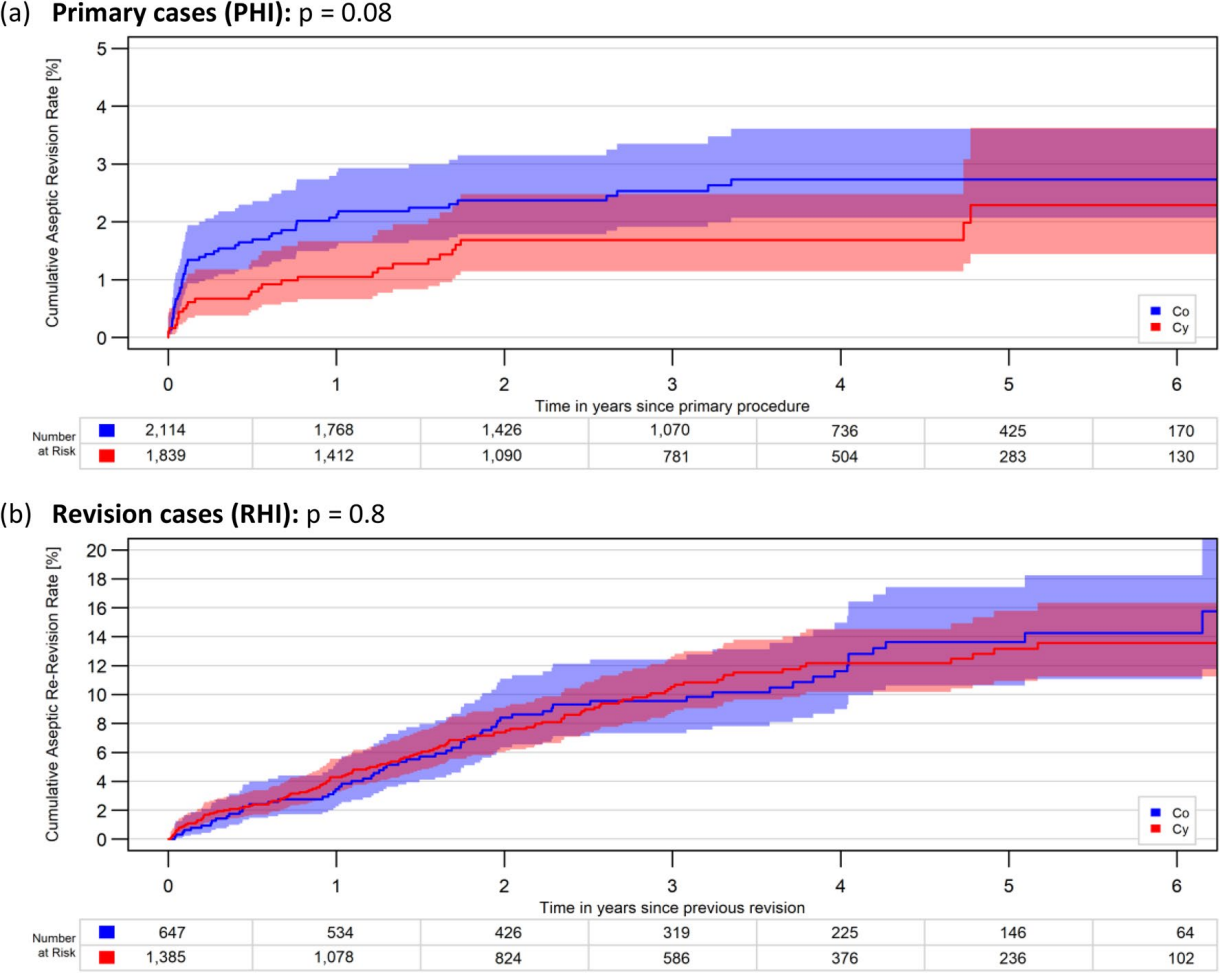
**a**, **b** Impact of stem conicity, all stems: 6-year cumulative aseptic revision rates of hinged implants with cemented tibial stems between Co and Cy stem designs in **a** primary cases (PHI) and **b** revisions cases (RHI)

With regards to stem conicity in overall, there were no significant differences of the 6Y-CARR, neither in the primary nor in the revision group: p = 0.08 and p = 0.8, respectively, (Fig. 1a, b).

### Stem diameter (only for Cy stems)

**Fig. 2:**
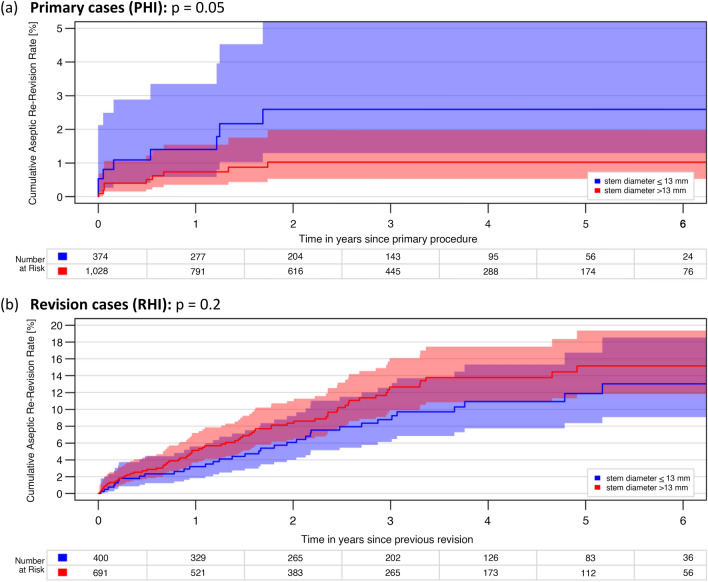
**a**, **b** Impact of stem diameter, only Cy stems: 6-year cumulative aseptic revision rates of hinged implants with cemented tibial stems between diameters ≤ 13 mm and > 13 mm in **a** primary cases (PHI) and **b** revisions cases (RHI)

The evaluation of stem diameter was limited solely to Cy stems. While the effect analysis on the 6Y-CARR divided in diameters with ≤ 13 mm and > 13 mm showed a statistically significant difference in the PHI group (p = 0.05), this could not be confirmed in the RHI group (p = 0.2), (Fig. 2a, b).

### Stem length (only Cy stems)

**Fig. 3:**
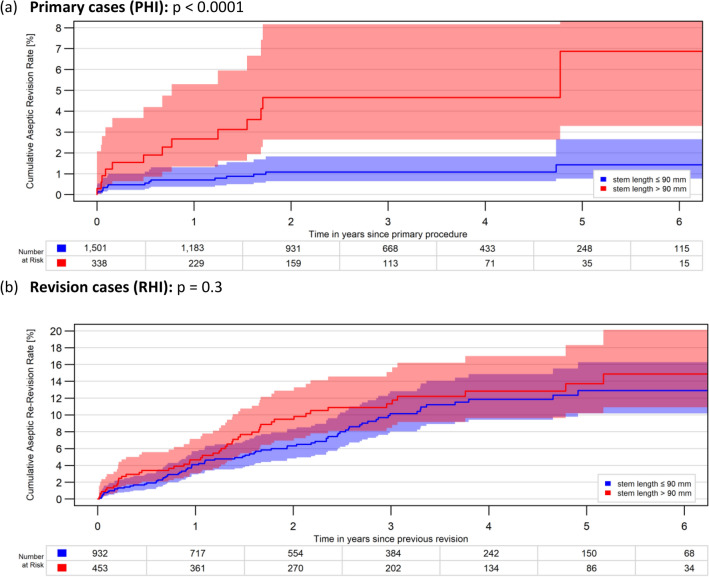
**a**, **b** Impact of stem length, only Cy stems: 6-year cumulative aseptic revision rates of hinged implants with cemented tibial stems between stem lengths ≤ 90 mm and > 90 mm in **a** primary cases (PHI) and **b** revisions cases (RHI)

Due to the limited sample size of the Co stems < 90 mm in both, PHI and RHI (n = 89 and n = 38, respectively), and in order to assure a good sample homogeneity, the statistical analysis of stem length was restricted on solely the cylindrical stem designs. The comparison revealed significantly better 6Y-CARR for the shorter stems ≤ 90 mm compared with stem lengths > 90 mm in the primary implantations (p < 0.0001). In contrast, no significant difference could be detected in the revisions (p = 0.3), (Fig. 3a, b).

### Stem offset (only Cy stems)

**Fig. 4:**
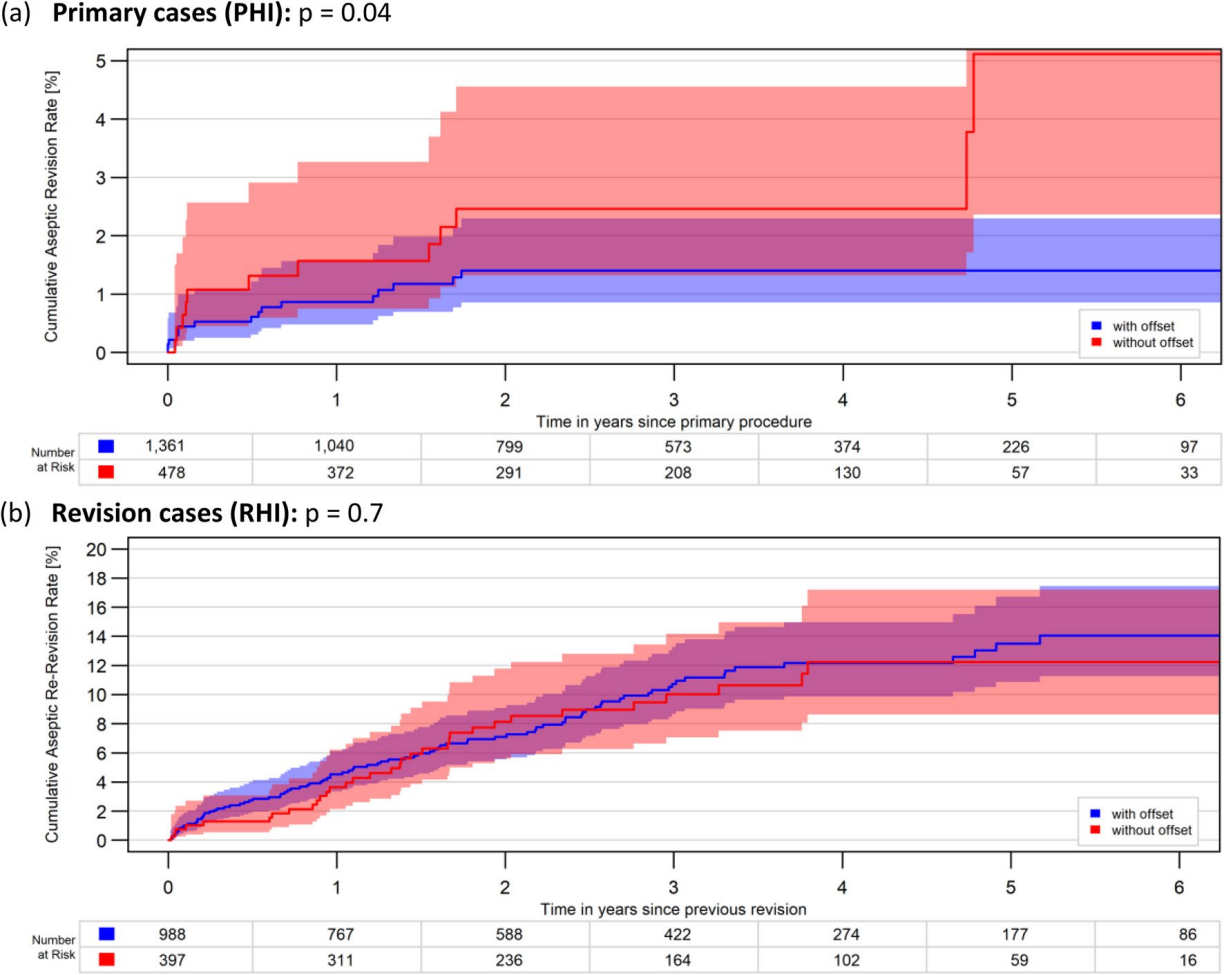
**a**, **b** Impact of stem offset, only Cy stems: 6-year cumulative aseptic revision rates of hinged implants with cemented tibial stems between stems with and without offset mm in **a** primary cases (PHI) and **b** revisions cases (RHI)

When dividing the Cy stems in those with offset and those without, a significant difference in CARR was detected in the PHI, with offset- stems being superior (p = 0.04) with a difference being more visible mostly after the 5th year. However, this effect was not observed in the RHI cases where both 6Y-CARR were similar (p = 0.7), (Fig. 4a, b).

## Discussion

### Main outcome of the study

Main outcome of the present registry study is that tibial stem conicity had in overall no significant impact on the 6-year aseptic survival rates of cemented revision total knee arthroplasty implants in the context of both, primary implantations and revisions. Therefore, the first hypothesis could be rejected.

Furthermore, cemented RTKA tibial stems with (1) diameter > 13 mm, (2) length ≤ 90 mm and (3) those with offset were associated with superior 6-year aseptic survival rates than their comparisons in the primary cases and therefore, the second hypothesis could also be rejected. However, the tendencies observed in the PHI group could not be observed further on in the more complex cases of the revision group, which in overall linked to worse survivorships with no differences between the stem design properties tested. This demonstrates the multifactorial dimension of RTKA failure beyond just implant design.

### Clinical trials and register-based current evidence

Available evidence on implant survivals and failure rates after RTKA with particular focus on aseptic loosening compiled by national or regional arthroplasty registries and related registry-based studies or representative clinical trials is summarized as follows (Table 3).

**Table 3:** Worldwide outcomes of revision total knee arthroplasty in terms of aseptic loosening rates (within all failure causes) and implant survivals (after an index revision or after a primary RTKA implantation) as reported by arthroplasty registries and related clinical studies, ordered alphabetically by countries

Countries	Source	Aseptic loosening^a^				Implant survivorship		
		SP	FU	Scenario	Rates	SP–EP	Scenario	C(A)RR; C(A)SR
US	CS [44]	All	5.4	RE	4.9%	All–AC		5 y-, 7.5 y-CASR: 82%, 78.2%
	CS [45]	AC	5; 7	RE	1.3–2.1%; 2.3–4.5%	AC–all	1st RE	5 y-, 10 y-, 15 y-CRR: 16.1%, 26%, 34.1%
Australian	AR’20 [2]	AC	15	1st RE	8%^c^ (33.3%)	AC–all	1st RE	5 y-, 10 y-, 15 y-CRR: 15.5%, 20.9%, 25.6%
	AR’22 [46]	AC	12	PI HI	0.8% (12.1%)	All–all	PR HI	5 y-, 7 y-, 10 y-CRR: 11.3%, 14.2%, 18.3%
Denmark	AR’21 [47]					All–all	1st RE	5 y-, 7 y-, 10 y-CRR: 11.3%, 14.2% and 18.3%
	RS [48]	All	3.2	1st RE	(31–33%)	All–all	1st RE	5 y-, 20 y-CRR: 16–18%, 19–23%
Netherlands	AR’22 [49]	All	7	1st RE	(24.2%)	All–all	1st RE	5 y-, 8 y-CRR: 15.7%, 18.9%
England, Wales and Northern Ireland	CS [13]	All	14	RE	4.9% (13.3%)	All–all	RE	10 y-CSR: 90.2%; 36.6%
	AR’22 [50]	All	18	RE	(33.4%)	All–all	RE	5 y-, 10y-CRR: 11.6%, 15.4%
Finland	RS [51]					All–all	1st RE	5 y-CSR: 89%; 5 y-CRR: 85–92%
	CS [52]	All		1st RE	(6.7%)	All–all	1st RE	10 y-CSR: 81.7%
Germany	Current RS					AC–AC AC–AC	PI HI RE HI	6-y CARR^b^: 2.5% 6-y CARR^b^: 14.7%
Italy	AR’22 [53]	All	6.1	RE	(33.7%)	All–all	RE	5 y-, 7 y-, 10 y-CSR: 87.2%, 84.9%, 82.9%
New Zealand	AR’20 [54]	All	2.4	1st RE	(22%)	All–all	1st RE	5 y-, 6 y-, 10 y-CSR: 83.8%, 82.7%, 78.8%
Norway	RS [10]	AC	4.5	RE	(26%*)*	AC–all	RE	5 y-, 7 y-, 10 y-CRR: 85%, 78%, 71%
Sweden	CS [55]	AC	8.8	RE	9.5%	AC–AC AC–all	RE	10 y-CSR: 89% 10 y-CSR: 65.1%
India	CS [56]	All	10.3	RE	9.1%	All–all	PI and RE	10 y-CSR: 90.7%

*CS* clinical study, *AR* annual report (of an arthroplasty registry), *RS* registry-based study, *SP* starting point, *EP* endpoint, *FU* follow up in years, *all* all causes, *AC* aseptic cases, *RE* revisions as starting point, *PI* primary implantation of revision implant as starting point, *HI* hinged implants, *y* year, *t* tibia, *f* femur, *C(A)RR* cumulative (aseptic) revision/re-revision rate, *C(A)SR* cumulative (aseptic) survival rate, *X%(Y%)* rate from all cases tested (rate within all revision causes)

^a^Aseptic loosening rates as occurred within all failure causes

^b^Aseptic re-revision causes that were less stem-associated (patella or soft tissue issues and solely line exchanges) were not considered

^c^Cumulative incidence

In a Finnish registry-based study on 2637 RTKA of all causes (1990–2002) Sheng et al. reported an overall 5-year survival rate of 89% after 1st revision. The re-revision rates were significantly higher when 1st revision was performed in less than 5 years after primary TKA (85% vs. 92%, p < 0.0005). Cement fixation (p < 0.005) and bone-grafting (p = 0.05) showed better survivals than hybrid and cementless fixations [51]. Hintze et al. analyzed the mid-term outcomes of 125 HI-RTKA with Cy stems that were performed between 2004 and 2013 in a Finnish high-volume arthroplasty center. 12% underwent re-revision, of which 40% due to aseptic failures including fractures and patella issues (6.7% solely aseptic loosening). The 10-year implant survival was 81.7% (all causes) [52].

In a Danish registry-based study comparing re-revision rates after 1st revision (a) due to “pain” without other pathologies (n = 1111) vs. (2) due to aseptic loosening (n = 2514), the 2-, 5- and 20-year cumulative re-revision rates were comparable for both groups: (a) 12%, 18%, 23% and (b) 11%, 16%, 19%. With a mean FU time of 3.4 years between 1st–2nd revision for (a) and 2.9 years for (b), aseptic loosening was the most common re-revision indication (31% and 33%) [48]. Finally, according to the Danish Arthroplasty Registry (DKR) annual report 2021, the 5-, 7-, and 10-year survival rates after 1st revision including all causes were 11.3%, 14.2% and 18.3%. The overall re-revision rate between 1st–2nd revision (20.2%) was considerable less than between 2nd–3rd revision (26.9%), which corresponds to the findings of other arthroplasty registers and indicates the increasing risk with every new revision added each time [47]

A Norwegian register study examined the failure rates of 1016 aseptic revisions (85% cemented). With a median follow-up of 4.5 years, re-revision was performed in 14.3%, whereby after excluding infections and patella causes the incidence rate dropped to 9.5%. Within all re-revision causes aseptic loosening occurred 17% on the tibia and 9% on the femur. The 5-, 7- and 10-year cumulative survival rates of index revisions (endpoint all reasons) were 85%, 78% and 71%, respectively. Type of fixation did not affect the survival rates of revisions [10].

In a Swedish single-center study, Gudnason et al. followed the implant survivals of a rotating hinge RTKA system with cemented conical stems. Including only aseptic index revisions as starting point he reported 9.5% aseptic loosening at a mean FU of 8.8 years. The 10-year survival rate with endpoint re-revision due to aseptic loosening was 89.2%, whereas due to any reason 65.1% [55].

The overall 5- and 8-year CRR after 1st revision by the Dutch Arthroplasty Register (LROI) Report 2007–2012 was 15.7% and 18.9%, respectively. Within 7 years after 1st revision, aseptic loosening occurred in 24.2% (tibial 16.3% and femoral 7.9%), which constitutes the 3rd most frequent cause after infection (37.1%) and instability (28.8%) [49].

According to the 2022 Report of the Regional Register of Orthopaedic Prosthetic Implantology (RIPO) in Emilia-Romagna Region (Italy), 98.8% of total revisions had a cemented fixation. At a mean FU of 6.1 years the revision failure rate (excluding issues with patella, spacer or soft tissues) was 13.1%. Within all failure causes aseptic loosening (total and partial) was 33.7% (9.6% tibial, 1.8% femoral and 22.3% on both sides). The 5-, 7- and 10-year revision survival rates were 87.2%, 84.9% and 82.9% [53].

In the United States, Mortazavi et al. (2011) reported in a single-center study on 499 index revisions, 20.4% revision failure (102 knees). Of all causes, aseptic loosening occurred in just 4.9%. The 5- and 7.5-year aseptic survival rates estimated were 82% and 78.2% [44]. In another high-volume single-center retrospective study on 1814 aseptic index revisions, Sierra et al. reported 373 re-operations. Aseptic loosening occurred in 1.3–2.1% and 2.3–4.5% at 5 and 7 years, respectively. The 5-, 10- and 15-year cumulative re-revision rates were 16.1%, 26%, and 34.1% [45].

In the Australian Orthopaedic Association National Joint Replacement Registry (AOANJRR) Annual Report 2020, aseptic loosening following 1st aseptic revision occurred in 5% presenting the most common cause of all re-revisions (33.3%) and reaching even higher ratings 40.5% if the 1st revision diagnosis was also aseptic loosening. The use of stem extension and a metaphyseal fixation improved the CRR of the 1st revisions. Use of a hinged RTKA by the 1st revision reduced the risk of re-revision due to instability. The 5-, 10- and 15-year CRR of 1st aseptic revisions were 15.5%, 20.9% and 25.6% respectively [2]. After removing all infections and patella causes as endpoint, the 15-year CRR after 1st aseptic revision dropped to 10.8%, which corresponds to the results of the present study (6Y-CARR 14.7%). In the AOANJRR Annual Report 2022 on survivals of RTKA implants in the setting of complex primary cases, the 5-, 7- and 10-year CRR (all diagnoses) for hinged RTKA models were 11.3%, 14.2% and 18.3%, respectively. Within all revision causes of PHI, aseptic loosening occurred in 12.1% [46]. When excluding infections and patella revisions, the overall aseptic revision rates after primary hinged implants (for osteoarthritis) could be estimated at about 2.3%, which is also comparable to our findings (6Y-CARR 2.5%) (Table 2a–b).

The 5-, 6- and 10-year re-revision free survivals (all causes) reported 2020 by the New Zealand Joint Registry (NZOA) were 83.8%, 82.7% and 78.8%, respectively. The mean time between 1st–2nd revision was 2.4 years with tibia loosening (11.9%) followed by femoral loosening (10.1%) being the third and fourth most frequent RTKA failure patterns [54].

The re-revision rates by the National Joint Registry (NJR) annual report 2022 at 5 and 10 years were 11.6% and 15.4%. Aseptic loosening (33.4%) was the most common reason for re-revision [50]. In a recent singe-surgeon study on a modular rotating hinge system with a combined stem conicity design (cylindrical-conical) that was used with cemented fixation mostly in complex revision cases (56.1% with ≥ 3 previous procedures), the overall 10-year survivorship was 90.2%. The revision failure rate was 36.6%. Aseptic loosening requiring re-revision occurred in 4.9% of index revisions (13.3% between all failure causes) [13].

In an Indian study, Rajgopal et al. examined the long-term results of a modular RTKA system with a Cy stem that was used in both, complex primary cases (n = 36) and revision cases (n = 81). Most often short cemented stems were advocated. If a long stem was necessary, a hybrid fixation technique was used. With a mean FU of 10.3 years aseptic loosening occurred in 9.1%. The overall 10-year survival rate (all reasons) was 90.7% [56].

### Rotating hinged versus semi-constrained design

In a current meta-analysis on RTKA outcomes, implant survivals of rotating hinged implants were superior than that of semi-constrained designs (SC) in short-terms < 5 years (87.4% vs. 75.0%). However, in the mid-term run (5–10 years) hinged implant survivals deteriorated and balanced with the SC (81.3% vs. 83.8%) [57]. This can be explained by the fact that although hinged implants may improve initially stability and likely contribute to superior short-term survivals compared with SC [58], they can also develop a higher risk for aseptic loosening in the midterm due to increased stresses transferred to the bone-cement interface and the stems [59]. Thus, our target was exactly this entity, focused on the mid-term survivals of hinged RTKA implants.

### Stem fixation

Cemented stem fixation compensates incongruences between cancellous bone and implant improving load distribution [60]. This may be why it provides excellent long-term results in RTKA [31, 35, 39, 61, 62]. One important drawback however, remains its challenging revision especially in case of an infected well-cemented long stem [27–29]. Thus, short stems with conical profile are preferred for cemented fixation. A multi-center study on 82 aseptic RTKA cases comparing cemented metaphyseal stems vs. diaphyseal press-fit stems reported equal incidences of aseptic loosening (4% vs. 3–6%) at 6–10 years of FU [63]. Furthermore, in another comparison of cemented fixation (53%) vs. press-fit fixation (47%) on 202 RTKA metaphyseal stems, aseptic loosening at 2-year FU occurred in 0% vs. 10%, respectively [36]. This outcome is supported also from finite-element studies. Thus, it could be demonstrated that cemented tibial stem extensions cause less micromotions in the bone-tibial tray interface and at the level of the tip of the stem than press-fit stems, which is associated with reduced risk of implant loosening [64].

### Stem conicity

A possible advantage of conical stem designs from a biomechanical point of view is that a cone-shaped cement coat could theoretically reduce shear stresses and micromotions in the longitudinal axis of the cement–implant interface, which in the long-term run could otherwise trigger bond failure and loosening. However, at the current state of knowledge, no clinical or biomechanical evidence exist to support this theoretical concept. Another advantage of conical stems lies in the easier removal, due to the short displacement required until complete detachment from the cement coat can occur [27–29].

A comprehensive review of the literature upon the impact of stem profile on RTKA outcomes was conducted for the first time in a recent study [18]: Involving 102 studies (45,963 cases), cemented fixation was used in all Co stems and in 11.6–43% of Cy stems. The RTKA implants were divided in primary and revision cases. In the first group of mainly primary cases (37,340 cases), aseptic loosening occurred in 3.3 ± 4.4% (mean FU 9.4 years) for the Co stems and 1.8 ± 4.2% (mean FU 6 years) for the Cy stems. In the present study, the 6Y-CARR of the PHI between Co and Cy were similar (2.7% and 2.3%, p = 0.08). In the second group of only revisions (8623 cases), the incidence of aseptic loosening was 5.1 ± 6.3% (mean FU 6 years) for the Co stem and 3.5 ± 5% (mean FU 5.2 years) for the Cy group. The incidence of radiological radiolucent lines was not included within the aseptic loosening. The re-revision rates were 18.3 ± 22.5% and 10.2 ± 5.9%, respectively. Equally, in the present study, the 6Y-CARR for the RHI was 13.5% (Co) and 15.8% (Cy) (Table 2a–b). However, it is necessary to consider the fact that while this study included cemented and cementless stems, the present study was focused only on cemented tibia stems.

### Stem diameter

In the context of press-fit or hybrid stem fixation, stem diameter is important in order to gain a sufficient canal filling ratio (CFR) [65, 66]. This principle doesn't apply for the cemented fixation technique. Stem diameter can therefore be downsized and still retain its high primary stability when cement is used. However, according to recent evidence on rotating hinged RTKA implants with cemented stems, a femoral canal wider than 19 mm, as measured 20 cm proximal to the joint line, is associated with increased risk for aseptic loosening [11, 67]. Furthermore, even if above observations concern the femoral side, it can be generally agreed from biomechanical point of view, that the higher the contact area between implant and cement through a larger stem diameter, the better the load distribution and implant stability.

Therefore, the superior 6Y-CARR results observed by the cemented tibial stems with larger diameters > 13 mm in the current investigation can be explained in a certain degree by above theory.

### Stem length

There is no clear evidence concerning which stem length and fixation technique is more appropriate in hinged RTKA. A current finite element study using the same hinged revision model (RT-PLUS Modular Rotating Hinge, Smith & Nephew) that combines stems with Cy profile, compared the stresses and micromotions between short stems (95 mm) vs. long stems (160 mm) in cemented and press-fit fixation techniques [68]. The cemented fixation reduced the stresses as well as the micromotions compared to the press-fit fixation. This effect was more pronounced among the long stems. The use of cement on short stems did not provide any significant difference. According to this finding, the probability of aseptic loosening and stem-end pain after a hinged RTKA might be higher after press-fit fixation of long stems.

### Stem offset

Modular offset stems in RTKA are important to address some surgical challenges during joint reconstruction, such as a component-induced impingement due to overhang or a malalignment of the joint line and the limp due to preexisting extraarticular deformities and finally a flexion–extension gab mismatch requiring adjustment of the joint line [69, 70].

Clinical evidence on hinged RKTA reflects the above advantages of offset design revealing very good late results. Rajgopal et al. (2020) examined a modular rotating hinged RTKA implant in complex primary (30%) and revision (70%) cases on 111 patients and reported 10-year survival rates of 90.65% [56]. The present study is also aligned with the literature showing a 6Y-CARR of 1.3% and 14% for primary and revision cases (Table 2a–b and Fig. 4a, b).

### Strength of the study

Despite the continuing efforts for improvement, the RTKA failure rates are still high. Within all failure patterns, aseptic loosening is the second most frequent after infection ranging from 20 to 50% [10, 44, 71–73]. Thus, there is an urgent need for further research towards more satisfying RTKA results and implant survivals.

Although there are several studies that assess the impact of stem design and fixation techniques on RTKA outcomes, stem conicity has not yet been systematically analyzed in this context. In a recent single-center study on complex revision cases, aseptic loosening occurred more often in Cy than in Co cemented stems. However, the overall incidence including also cementless stems did not defer between the two profile groups. Furthermore, there was no assessment of implant survivorships [18].

Finally, as the existing studies are burdened with cohort inhomogeneity, lack of values about the exact stem design and a discrepancy in definitions of failure and re-revision rates, it is very difficult to extract reliable information from the literature.

This is the first large scale analysis based on national-wide data collections from the German Arthroplasty Registry, that examines the effect of different stem design properties such as conicity, diameter, length and offset on the 6-year aseptic survival rates of hinged RTKA systems with cemented tibial stems.

### Limitations

Some important aspects and limitations of the study are numbered as follows: (1) The results represent survivals of cemented tibial RTKA extensions only and not of all stems. However, as several studies in RTKA implants illustrate equal to higher failure rates on the tibial side, it was legitime to test solely tibial stems aiming improved homogeneity. (2) There were some missing values and documentation errors concerning for example the true number of cones or sleeves used. Thus, as metaphyseal fixation is well documented in the literature to improve implant longevity [11, 74–76], this can be regarded as potential bias factor. However, the cases involving cones/sleeves were very few to have a considerable effect on the test (0.2% in PHI and 3.9% in RHI). (3) We included all hinged RTKA implants independent of some considerable design differences (rotating or rigid) that may have also an impact on implant survivals [76]. However, failure patterns at the level of constrain mechanism are more often found in association with femoral stem loosening and not tibial [11, 40, 77–80]. Furthermore, the RTKA survivals found in the present study apply only for hinged implants and not for other levels of constrain such as PS or SC designs. (4) No patient-related risk factors could be considered. (5) In the RHI groups, the starting point was not differentiated based on the number of previous revisions and the complexity of index revision. (6) Not all utilization-scenarios that may involve within the diagnosis codes of a national registry data base can be further differentiated. Therefore, some highly complex cases leading to extreme outliers could potentially also influence the overall outcomes. (7) In certain group comparisons there was a considerably disproportionate distribution of the stem design properties tested within the included manufacturers, mostly concentrated in few well-established products. This may have led just as well to a disproportionally stronger system effects of some products against others. (8) The use of the planning software MediCAD^®^ to define the conical stem profile might involve some user-variations and thus potential discrepancies between measurements and the true manufacturer’s values. (9) Combined stem designs with conical proportions ≤ 30% of stem length were included in the Cy group, which might also have a (minor) influence on the results. Finally, (10) in order to examine the survival rates that are mostly associated with stem design, other revision causes such as infections, tumors, patella issues or soft tissue complications were excluded. Therefore, a direct comparison with the available literature and other registries is not possible and the results must be interpreted with caution.

### Interpretation and conclusions

The present study could demonstrate in overall a moderate effect of the tibial stem design on the mid-term aseptic survival rates of cemented RTKA. The impact of some stem design properties tested was significant in the primary cases but disappeared in the more complex and heterogenous revision settings. Given also the limitations of different system effects and utilization scenarios, interpretation of the present results must be done with great caution. Finally, as implants with equal survivals but at same time friendlier revisability are preferable, conical stem designs may constitute an advantage for the cemented hinged RTKA.

## Data Availability

The data that support the findings of this study are available from the authors but restrictions apply to the availability of these data which are property of the German Arthroplasty Registry EPRD (EndoProthetikRegister Deutschland) and their use was under application license from the EPRD gGmbH for the current study, and so are not publicly available. Data are, however, available from the authors upon reasonable request and with permission from the Data Protection Office German Arthroplasty Registry EPRD gGmbH, http://www.eprd.de.
